# Phenotypic Refinement of *ESAM* ‐Related Tight‐Junctionopathy: Novel Genetic and Ocular Findings and Literature Review

**DOI:** 10.1002/mgg3.70245

**Published:** 2026-06-09

**Authors:** Mauro Lecca, Chiara Bosetti, Federico Ruoli, Liviana Fontanel, Marco Mazza, Enza Maria Valente, Roberta Battini, Edoardo Errichiello

**Affiliations:** ^1^ Department of Molecular Medicine, Unit of Medical Genetics University of Pavia Pavia Italy; ^2^ Department of Developmental Neuroscience IRCCS Stella Maris Foundation Pisa Italy; ^3^ Department of Clinical and Experimental Medicine University of Pisa Pisa Italy; ^4^ Pediatric Ophthalmology Unit ASST Grande Ospedale Metropolitano Niguarda Milan Italy; ^5^ Developmental Neuro‐Ophthalmology Unit IRCCS Mondino Foundation Pavia Italy; ^6^ European Reference Network on Eye Diseases (ERN‐EYE), ASST Grande Ospedale Metropolitano Niguarda Milan Italy; ^7^ Unit of Neurogenetics IRCCS Mondino Foundation Pavia Italy

**Keywords:** electroretinogram (ERG), *ESAM*, familial exudative vitreoretinopathy (FEVR), retina, tight junction (TJ), whole exome sequencing (WES)

## Abstract

**Background:**

Endothelial cell‐selective adhesion molecule (*ESAM*) is a tight junction protein essential for blood–brain barrier integrity and angiogenesis. Bi‐allelic loss‐of‐function variants in *ESAM* cause NEDIHSS (“Neurodevelopmental disorder with intracranial hemorrhage, seizures, and spasticity”), a neurodevelopmental/neurovascular disorder with antenatal/neonatal onset, characterized by global developmental delay/intellectual disability (GDD/ID), epilepsy, spasticity, ventriculomegaly, and intracranial hemorrhages. Ocular involvement, particularly retinal vascular anomalies, has been variably reported.

**Methods:**

A multidisciplinary team of pediatric neurologists, ophthalmologists, and clinical geneticists evaluated the proband through clinical assessment, neuro/ocular imaging, and whole‐exome sequencing.

**Results:**

We describe a 3‐year‐old male from consanguineous Albanian parents carrying a novel homozygous splice‐site *ESAM* variant (c.70+1G>T). He presented with GDD, seizures, and brain imaging abnormalities consistent with NEDIHSS. Remarkably, bilateral optic nerve hypoplasia, esotropia, retinal detachment, absent electroretinogram response, and structural eye anomalies, including left eyeball hypoplasia and iris displacement, were observed. Review of our and previous cases indicates that 45% (10/22) of NEDIHSS individuals present ocular manifestations, mainly retinal vascular defects, reinforcing the emerging role of *ESAM* in retinal endothelial integrity.

**Conclusions:**

This case broadens the mutational and clinical spectrum of *ESAM*‐related disease, underscores the need for detailed ocular evaluations in NEDIHSS, and supports inclusion of retinal anomalies within its core phenotype.

## Graphical abstract

Overview of neurological, ocular, and genetic findings in individuals with bi‐allelic loss‐of‐function (LoF) *ESAM* variants. All affected subjects (*n* = 21) exhibited characteristic neurovascular and neurodevelopmental anomalies, while 45% also showed ocular (mainly retinal) involvement. Exome sequencing identified a novel bi‐allelic splice‐site *ESAM* variant (c.70+1G>C) in the newly reported individual.

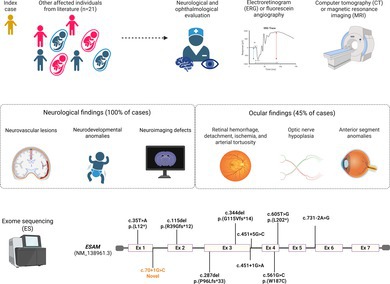

## 1 Introduction

*ESAM* (OMIM *614281) encodes a tight junction (TJ) protein related to junctional adhesion molecules (JAMs), which contribute, in synergy with claudins (especially claudin‐5) and occludin, to the formation and maintenance of the blood–brain barrier (BBB), the core structure of the neurovascular unit (NVU). In mice, complete loss of Esam expression dramatically impairs angiogenesis (Ishida et al. 2003).

We previously described 13 individuals (including four fetuses) with *ESAM* bi‐allelic loss‐of‐function (LoF) variants and identified a novel rare disease trait named NEDIHSS syndrome (“Neurodevelopmental disorder with intracranial hemorrhage, seizures, and spasticity”—OMIM #620371), a tight‐junctionopathy with antenatal/neonatal onset showing global developmental delay/intellectual disability (GDD/ID) with absent or severely delayed speech, epilepsy, varying degrees of spasticity, ventriculomegaly, and intracranial hemorrhages as cardinal clinical signs (Lecca et al. 2023). Other notable phenotypic features were cerebral calcifications, focal white matter lesions, dilation of lateral ventricles, hydrocephalus, thin corpus callosum, and variable microcephaly (Lecca et al. 2023).

In the original cohort of subjects, ocular findings were observed in 5/13 (39%) cases, consisting mainly of vascular anomalies of the retina, such as retinal ischemia (4/5), retinal hemorrhage (2/5), arterial tortuosity (2/5), fibrovascular proliferation (2/5), and retinal detachment (1/5) (Lecca et al. 2023). Since then, additional cases of NEDIHSS have been described, broadening the range of symptoms, including ocular manifestations: an individual from Saudi Arabia presented with optic atrophy, exotropia, and a negative electroretinogram (ERG) (Alshammari et al. 2024); two Egyptian probands exhibited a pale optic disc (Abdel‐Salam et al. 2025); and a recently reported Turkish proband showed bilateral cataracts (Alomari et al. 2026).

In this study, we investigated a new individual carrying a novel homozygous *ESAM* variant and refined the NEDIHSS phenotype, with a focus on ocular/retinal involvement. A comprehensive literature review is also provided.

## 2 Results

### 2.1 Clinical and Genetic Findings

The proband is a 3‐year‐old male, second‐born to healthy consanguineous Albanian parents, by spontaneous delivery with normal neonatal parameters (weight 3200 g, 28 pct −0.58 SD; length 49 cm, 33 pct −0.44 SD). He manifested global developmental delay (GDD) characterized by motor (poor head control, repetitive/spastic movements, axial hypotonia) and cognitive impairment (severe speech delay). The constellation of neurological symptoms detected in the proband was consistent with previously observed NEDIHSS features and included tonic–clonic seizures (onset at 5 months, the initial reason for referral), cortical atrophy and dilation of lateral ventricles with subependymal calcifications (detected by brain computed tomography (CT) scan at 5 months of age). Brain magnetic resonance imaging (MRI) revealed hydrocephalic dilation of the lateral ventricles with hemosiderin deposition (suggesting previous intracranial hemorrhages), cerebral white matter and corpus callosum hypoplasia, and brainstem hypoplasia due to thinning of the pyramidal tract. Remarkably, during the first year of life, the proband also developed talipes equinovarus (bilateral, with right‐sided predominance), respiratory gurgling, and auditory hypersensitivity.

#### 2.1.1 Ocular Findings

At 3 months of age, a first ophthalmological evaluation detected bilateral optic nerve hypoplasia, sensory esotropia, retinal detachment and blindness of the left eye. At 5 months, visual evoked potentials (VEP) and ERG were performed, confirming poorly reproducible responses, reduced amplitude bilaterally, and absent ERG response bilaterally, suggesting impaired retinal‐based visual conduction. Ophthalmological re‐evaluation at 9 months highlighted a reduced bilateral pupillary reflex, due to anterior displacement of the iris‐lens diaphragm, and a small, subatrophic optic disc with peripapillary scarring in the right eye. Additional ocular manifestations included hypoplasia of the left eyeball, along with spherophakia and nystagmus.

**FIGURE 1:**
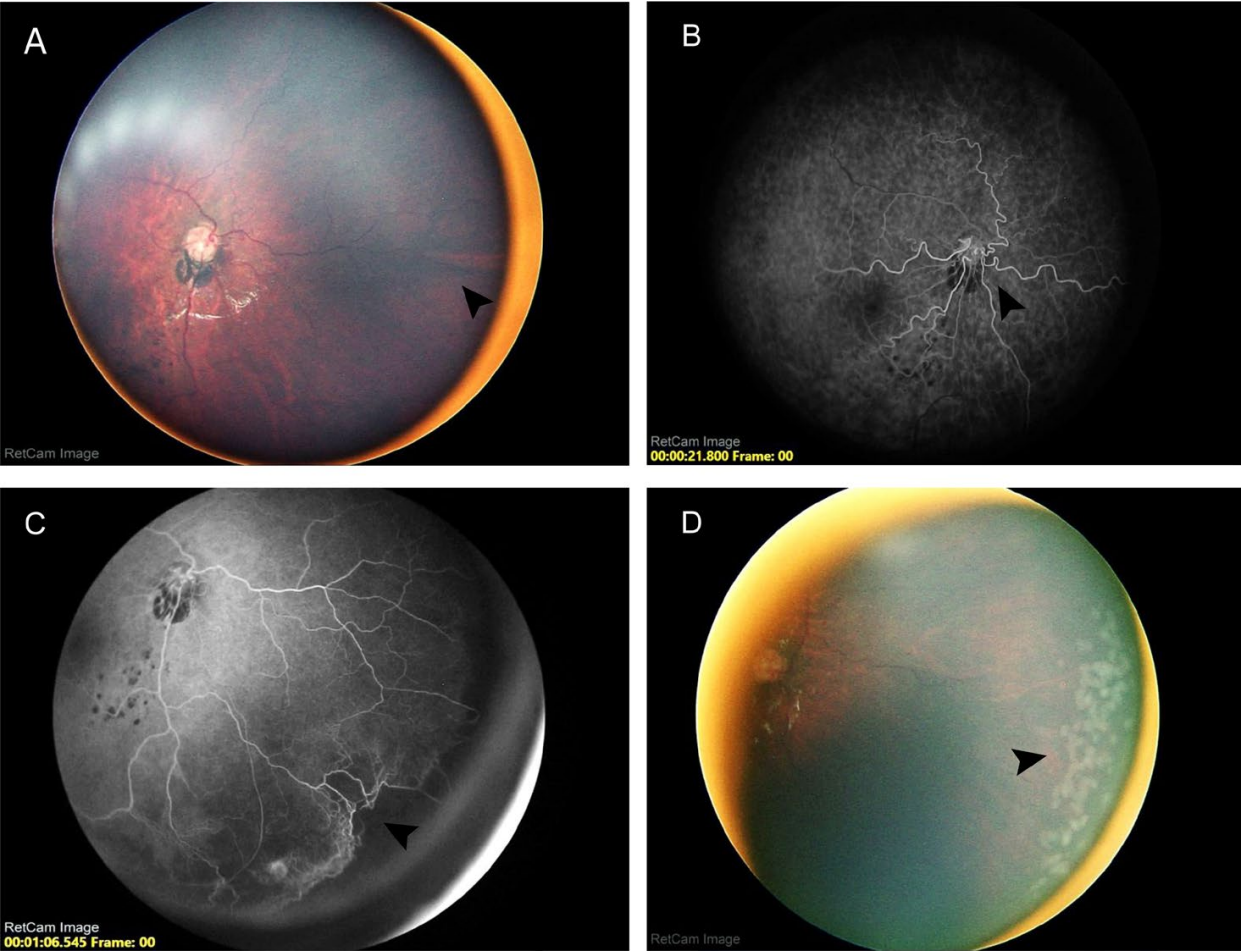
Fundus examination and fluorescein angiography images on the proband's right eye. (A) Wide‐field fundus pictures of the eye showing a dystrophic‐appearing retina with scattered pigmented deposits in the posterior pole, vascular thinning, tortuosity and peripheral avascularity. (B, C) Fluorescein angiography showing peripheral avascularity with vascular tortuosity and ischemic areas; fluorescein angiography images were captured through a 130° lens objective without magnification. (D) The eye was treated with argon laser photocoagulation on the peripheral ischemic retina with a circumferential treatment, performing 2260 spots with 190 mW power and 150 ms pulse duration settings. All the images have been taken with RetCam Image.

At 3 years, ocular assessment with an ophthalmic surgical microscope was performed under sedation. The anterior segment of the right eye was normal, the cornea was transparent, the anterior chamber was deep, the pupil was circular, and the lens was transparent and in situ, with no signs of donesis. Right eye evaluations included the fundus examination, which revealed the subatrophic and dysmorphic optic nerve, and pigment deposits were found in the peripapillary area and in the posterior pole. The retinal blood vessels were thinned and tortuous, and a circumferential avascular zone was found in the periphery and mid‐periphery regions of the retina (Figure 1). Fluorescein angiography confirmed these findings (Figure 1) and revealed the presence of immature vascular proliferation on the edges. To minimize the risk of retinal detachment (as occurred in the left eye), circumferential argon laser photocoagulation was performed on the right avascular retina. The left eye appeared hypoplastic due to the previous total retinal detachment and consequent *phthisis bulbi*. The ophthalmologic assessment was conducted following current best practices for pediatric retinal disorders, in line with established European reference standards (ERN‐EYE recommendations) (Lorenz et al. 2023).

#### 2.1.2 Genetic Findings

Array‐comparative genomic hybridization (CGH) and targeted next‐generation sequencing (NGS) epilepsy panel were negative. On the other hand, whole‐exome sequencing identified a homozygous splice‐site variant in *ESAM*: NM_138961.3:c.70+1G>T. The variant was predicted by multiple *in silico* tools (e.g., HSF, MaxEntScan, SpliceAI, Pangolin) to disrupt the highly conserved donor splice‐site of exon 1, whereas only five heterozygous individuals (5/1611040, 0.0003104%)—and no homozygotes—are reported in gnomAD v.4.1.0. Therefore, according to the ACMG/AMP and ACGS guidelines, the variant was classified as pathogenic (PVS1, PM2). Functional validation of the variant at the RNA level could not be performed due to the unavailability of additional samples from the proband. Sanger segregation analysis confirmed the presence of the heterozygous c.70+1G>T variant in both parents. A further analysis by AutoMap (Quinodoz et al. 2021) revealed several regions of homozygosity (ROHs) for a total of 63.61 Mb, confirming parental consanguinity (coefficient of inbreeding 1/64, corresponding to second's degree relationship) and including a ~4.4 Mb ROH (runs of homozygosity) encompassing *ESAM* (chr11:123,058,226‐127,462,983; GRCh38/hg38). The variant has been submitted to ClinVar (ID: VCV004086789.1).

### 2.2 Literature Review and Phenotypic Comparison

To date, nine different homozygous *ESAM* variants (7 pathogenic LoFs: c.115del, c.287del, c.35T>A, c.451+1G>A, c.344del, c.731‐2A>G, c.60T>G; one missense and a splice‐site with uncertain significance (VUS): c.561G>C, c.451+5G>C) have been reported in 21 individuals from four different studies (Lecca et al. 2023; Alshammari et al. 2024; Abdel‐Salam et al. 2025; Alomari et al. 2026), all sharing neurodevelopmental and neurovascular features (Table S1).

Beyond the canonical manifestations, ocular (mainly retinal) anomalies, namely retinal detachment (HP:0000541), retinal vascular (arterial/venous) tortuosity (HP:0012841), retinal hemorrhage/ischemia (HP:0000573), and cataract (HP:0000518), were observed in a large portion of NEDIHSS subjects (10/22, 45%) included both in our first study (individual 1 from pedigree 1, and siblings 2 and 3, from pedigree 2) and in subsequent studies (individuals 3 and 4 (Abdel‐Salam et al. 2025); individual 2 (Alomari et al. 2026); index case (Bayramoğlu et al. 2023)).

In most cases, the ophthalmological features were not investigated in depth, with the sole exception of ‘individual 3’ described by Lecca *et al.* (2023), whose ophthalmological history was further refined by Bayramoğlu *et al.* (2023), highlighting nystagmus and profoundly delayed arteriovenous transit (> 90 s) with incomplete venous filling as detected through fluorescein angiography.

The ocular findings of the current proband are comparable and resemble the condition known as Familial Exudative Vitreoretinopathy (FEVR).

## 3 Discussion

The implication of *ESAM* loss in retinal damage is not surprising considering the essential role of the encoded TJ protein in maintaining endothelial cells' permeability and its extreme abundance in retinal blood vessel endothelial cells (Human Protein Atlas: 566.1 nTPM). Similar retinal manifestations are observed in three other systemic conditions closely related to NEDIHSS neurovascular phenotypes, namely *JAM3* tight‐junctionopathy and *COL4A1*/*COL4A2* collagenopathies (Table 1). Furthermore, heterozygous *de novo* variants in *CLDN5*, encoding claudin‐5, a TJ protein highly expressed in BBB endothelial cells, have been recently identified in 15 individuals showing neurological and vascular phenotype (seizures, microcephaly, brain calcifications, and NDD) overlapping with NEDIHSS/tight‐junctionopathies (Deshwar et al. 2023). Specifically, 4 out of 15 individuals with *CLDN5* variants manifested ocular features, namely pale optic disc and cortical visual impairment.

**TABLE 1:** Ocular manifestations in tight‐junctionopathies and collagenopathies phenotypically comparable with NEDIHSS.

Ocular component	Ocular manifestation	Gene							
		*ESAM*		*JAM2*	*JAM3*	*OCLN*	*CLDN5*	*COL4A1*	*COL4A2*
		Current study	Literature						
Retina	Retinal hemorrhage	No	Yes (Lecca et al. 2023)	No	Yes (Kozak et al. 2022)	No	No	Yes (Meuwissen et al. 2015; Gasparini et al. 2024)	No
	Retinal detachment	Yes	Yes (Lecca et al. 2023)	No	No	No	No	Yes (Meuwissen et al. 2015; Gasparini et al. 2024; Coupry et al. 2010)	No
	Retinal arterial/venous tortuosity	Yes	Yes (Lecca et al. 2023; Abdel‐Salam et al. 2025)	No	Yes (Kozak et al. 2022)	No	No	Yes (Meuwissen et al. 2015; Gasparini et al. 2024)	Yes (Gasparini et al. 2024)
	Retinal ischemia	Yes	Yes (Lecca et al. 2023)	No	No	No	No	No	No
Optic nerve	Optic nerve hypoplasia	Yes	Yes (Alshammari et al. 2024)	No	No	No	No	Yes (Lecca et al. 2024; Dahl et al. 2020)	Yes (Dahl et al. 2020)
	Optic atrophy	No	Yes (Alshammari et al. 2024)	No	No	No	No	Yes (Meuwissen et al. 2015)	Yes (Meuwissen et al. 2015; Gasparini et al. 2024)
	Optic disc anomalies	No	Yes (Abdel‐Salam et al. 2025) (pale optic disc)	No	Yes (Kozak et al. 2022; Mochida et al. 2010) (pale optic disc)	No	Yes (Deshwar et al. 2023)	Yes (Gasparini et al. 2024)	Yes (Gasparini et al. 2024)
Iris	Iris anomalies	Yes	Yes (Lecca et al. 2023) (coloboma)	No	No	Yes (Slee et al. 1999) (iridolenticular adhesions)	No	Yes (Meuwissen et al. 2015; Gasparini et al. 2024; Coupry et al. 2010)	No
Lens	Cataract/lens morphological anomalies	Yes (spherophakia)	Yes (Alomari et al. 2026) (cataract)	No	No	No	No	No	No
Others	Strabismus	Yes	Yes (Alshammari et al. 2024) (severe)	Yes (Schottlaender et al. 2020) (exotropia)	No	No	No	Yes (An et al. 2021) (exotropia)	Yes (Duan et al. 2025)
	Nystagmus	Yes	Yes (Lecca et al. 2023; Bayramoğlu et al. 2023)	Yes (Schottlaender et al. 2020)	Yes (De Rose et al. 2021)	No	No	Yes (Meuwissen et al. 2015; Chalipat et al. 2024)	Yes (Bakhtiari et al. 2021)

The current proband (i.e., the 22nd individual with NEDIHSS syndrome) showed optic nerve hypoplasia, which has been rarely reported in NEDIHSS‐affected individuals (Alshammari et al. 2024), and left spherophakia, which has never been observed in NEDIHSS. Interestingly, optic nerve hypoplasia has been reported in a few individuals carrying pathogenic variants in *COL4A1* and *COL4A2* (Lecca et al. 2024; Dahl et al. 2020)—encoding type IV collagen alpha‐1 and alpha‐2 chain proteins (two vascular basement membrane proteins highly expressed in blood vessels that contribute to BBB maintenance), which cause intracerebral hemorrhage, porencephaly, cystic brain lesions, hydrocephalus, seizures, and retinal arterial tortuosity (BSVD1, OMIM #175780; BSVD2, OMIM #614483) (Table 1). Although no deleterious variants in the spherophakia‐related genes (HP:0034375) were identified in the proband, and cataracts (bilateral) have already been reported among the possible manifestations of NEDIHSS, further reports are needed to determine whether spherophakia represents a phenotypic expansion of NEDIHSS syndrome. At present, this finding may represent a novel feature pending replication, and any causal relationship between *ESAM* loss and the observed lens morphology remains speculative.

In addition, despite lacking a standardized ophthalmological evaluation across all the NEDIHSS‐affected subjects reported to date worldwide, other ocular anomalies frequently observed in *ESAM* individuals (occurring either in combination with retinal anomalies or isolatedly) such as optic atrophy, esotropia, iris coloboma, pale optic disc, cataract, and nystagmus have also been reported in other tight‐junctionopathies (Table 1).

In conclusion, we reported a new individual affected by NEDIHSS syndrome caused by a novel homozygous LoF/splice‐site variant in *ESAM*, expanding the mutational spectrum. Furthermore, our findings show that retinal anomalies, such as retinal detachment, ischemia, hemorrhage, and arterial/venous tortuosity, may be detected in about half of subjects with NEDIHSS, thus underlying careful ophthalmological evaluation as a crucial step in the diagnostic and therapeutic workup of NEDIHSS probands. Finally, we recommend including *ESAM*, as well as other TJ genes with supporting evidence for retinal expression/phenotype, among genes related to retinal disorders (currently not included in major panels such as PanelApp version 8.10).

## Author Contributions

M.L.: data curation, formal analysis, investigation, writing – original draft, writing – review and editing; C.B.: investigation, writing – review and editing; F.R.: investigation, writing – review and editing; L.F.: investigation, writing – review and editing; M.M.: funding acquisition, writing – review and editing; E.M.V.: funding acquisition, writing – review and editing; R.B.: funding acquisition, writing – review and editing; E.E.: conceptualization, data curation, formal analysis, funding acquisition, investigation, writing – original draft, writing – review and editing, project administration, supervision.

## Funding

This work was partly funded by the Italian Ministry of Health (Ricerca Corrente 2022–2024) granted to IRCCS Mondino Foundation and IRCCS Stella Maris Foundation.

## Consent

A signed informed consent form was obtained from the proband's parents permitting the use of the data for educational/research purposes and for publication of the case details.

## Conflicts of Interest

The authors declare no conflicts of interest.

## Supporting information


**Table S1:** Demographic, genetic, and clinical findings in individuals carrying homozygous *ESAM* variants.

## Data Availability

The data that support the findings of this study are available from the corresponding author upon reasonable request.
